# A laboratory-based, low-energy, multi-modal x-ray microscope with user-defined resolution

**DOI:** 10.1063/5.0082968

**Published:** 2022-06-08

**Authors:** Michela Esposito, Lorenzo Massimi, Ian Buchanan, Joseph D. Ferrara, Marco Endrizzi, Alessandro Olivo

**Affiliations:** 1 Department of Medical Physics and Biomedical Engineering, University College London, Malet Place, Gower Street, London WC1E 6BT, United Kingdom; 2 Rigaku Americas Corporation, 9009 New Trails Drive, The Woodlands, Texas 77381, USA

## Abstract

We report on the development of a low-energy x-ray phase-based microscope using intensity-modulation masks for single-shot retrieval of three contrast channels: transmission, refraction, and ultra-small-angle scattering or dark field. The retrieval method is based on beam tracking, an incoherent and phase-based imaging approach. We demonstrate that the spatial resolution of this imaging system does not depend on focal spot size nor detector pixel pitch, as opposed to conventional and propagation-based x-ray imaging, and it is only dependent on the mask aperture size. This result enables the development of a multi-resolution microscope where multi-scale samples can be explored on different length scales by adjusting only the mask aperture size, without other modifications. Additionally, we show an extended capability of the system to resolve periodic structures below the resolution limit imposed by the mask apertures, which potentially extends dark-field imaging beyond its conventional use.

Molecular cell biology is moving toward functional studies of single cells in model organisms creating a need for multi-scale imaging of unstained mm-sized samples, which are unsuitable for optical imaging due to light scattering in thick samples. Similar requirements arise from, e.g., the increased interest in three-dimensional virtual histology of biopsies. X-ray phase-based imaging of mm-thick biological samples with *μ*m and sub-*μ*m resolution has the potential to be a valuable tool for bio-medical research due to the inherently higher contrast for soft tissue compared to conventional x-ray imaging.^1^ A number of phase-based imaging systems suitable for laboratory setups have been proposed over the past years based on different concepts, including free-space propagation,^2^ edge-illumination,^3–5^ speckle tracking,^6^ and Talbot–Lau interferometry.^7^ While excellent results have been obtained with propagation-based phase contrast imaging,^8^ so far a multi-contrast (phase, attenuation, and dark-field), low-energy x-ray system suitable for imaging small-size soft-tissue samples at high resolution was not available.

Here, we present a phase-based low-energy x-ray microscope using intensity-modulation masks and investigate its resolution limits. The masks shape the beam into an array of one-dimensional beamlets, which are tracked for the modifications imposed by the sample that allow to infer complementary sample properties.

The approach used is similar to edge-illumination^3–5^ but employs a high-resolution pixelated detector fully sampling the shaped beamlets (beam tracking^9,10^). The first three moments of the shaped beamlets are obtained from a single frame (single-shot retrieval) following:^10,11^ amplitude, mean, and variance of each beamlet are extracted with ( $A_{s},\mu_{s},\sigma_{s}^{2}$) and without the sample in place ( $A_{r},\mu_{r},\sigma_{r}^{2}$). Transmission (*T*), refraction (*R*), and (ultra-small-angle) scattering (*S*) can be calculated as follows:

$$T=\frac{A_{s}}{A_{r}},$$ (1)

$$R=\frac{\mu_{s}-\mu_{r}}{z_{3}},$$ (2)

$$S=\frac{\sigma_{s}^{2}-\sigma_{r}^{2}}{z_{3}^{2}},$$ (3)

where *z*_3_ is the sample-to-detector distance.

The microscope consists of a rotating anode x-ray generator, doubly curved multi-layer optics selecting the Cu K_*α*_ lines (8.05 and 8.04 keV selected with approximately a 1% resolution)^12^ and focusing the beam to a 350 *μ*m focal spot, an intensity-modulation mask, and a detector. The mask, fabricated by Microworks GmbH (Karlsruhe, Germany), is a substrate-free periodic gold structure ( ≥30 *μ*m thick) with 2 *μ*m wide apertures (slits) and a 19 *μ*m period. The detector consists of a scintillator, an objective, and a scientific CMOS camera, leading to an effective pixel pitch of 1.1 *μ*m and a 1.6 × 1.6 mm^2^ field of view. A schematic of the experimental setup is shown in Fig. 1. The mask was placed approximately 2.2 m downstream of the source (z_1_), and the samples were placed 10 mm away from the mask (z_2_), while the sample-to-detector distance (z_3_) was varied between 15 and 35 mm. The setup was optimized to meet two criteria: (a) the beamlets are adequately sampled so that their moments can be estimated; (b) the projected focal spot size is smaller than the mask periods so that each beamlet is independent from each other, avoiding excessive crosstalk. While both criteria have a dependency on source focal spot, the first condition also depends on the detector pixel pitch, mask aperture width, and propagation distance z_3_. The latter depends on both z_1_ and z_3_ as well as mask period. A more detailed discussion on the optimization of the microscope setup and an exploration of the parameters space is reported in Ref. 13.

**FIG. 1.**
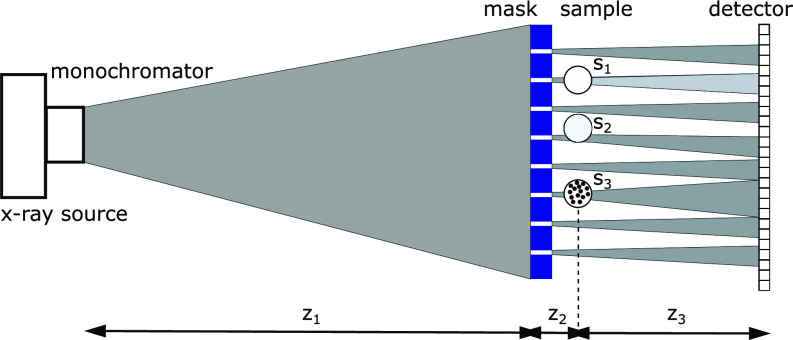
A schematic of the experimental setup. Effects on the shaped beamlets of attenuating (s_1_), refracting (s_2_), and scattering (s_3_) samples are pictorially represented.

The samples were scanned (dithered) along the direction of phase sensitivity (i.e., orthogonal to the mask slits) for a distance equal to the mask period, to achieve full sample illumination. To avoid aliasing, samples were dithered in steps ≤w/2, where *w* is the mask aperture. Images were acquired at 19 dithering steps with a step size of 0.95 *μ*m. At each dithering step, a number of 50 exposures of 10 s each were acquired and, after dark correction and pixel equalization, averaged before retrieval. At the end of a scan, a dataset without the sample in place was acquired to provide reference beamlets moments. It is worth noting that this technique can be considered single-shot in the absence of the dithering process, i.e., when the resolution is limited by the mask period. When aiming for aperture-driven resolution, however, a number of images proportional to the ratio of mask aperture to period are needed for retrieval. Although beam tracking has been shown suitable for tomographic reconstruction,^14^ in the current development stage, the microscope is operated in the 2D mode.

To quantify the spatial resolution of the system, we used a resolution target consisting of three regular patterns with 5, 2.5, and 1 *μ*m wide and 500 nm-thick gold bars, placed on a 200 nm-thick SiN_3_ membrane. The resolution target was imaged with and without the intensity-modulation mask in place, i.e., switching from beam tracking to propagation-based phase-contrast imaging. Figure 2 shows the pattern with 2.5 *μ*m bars imaged at different sample to detector distances (z_3_) in propagation-based imaging (a) and in beam tracking (b); the retrieved refraction channel is shown for the latter. All images are accompanied by the corresponding intensity profiles. In the propagation-based mode, increasing z_3_ leads to a loss of spatial resolution resulting in a reduced modulation at z_3_ = 25 and 35 mm, arising from the increase in projected focal spot size (penumbra blurring)^15^ with z_3_, which is 2.4, 4.0, and 5.6 *μ*m at the three propagation distances used, deliberately straddling the size of the imaged detail. Conversely, in beam tracking, the measured modulation for the 2.5 *μ*m feature size appears largely unaltered by the increase in z_3_ [Fig. 2(b)].

**FIG. 2.**
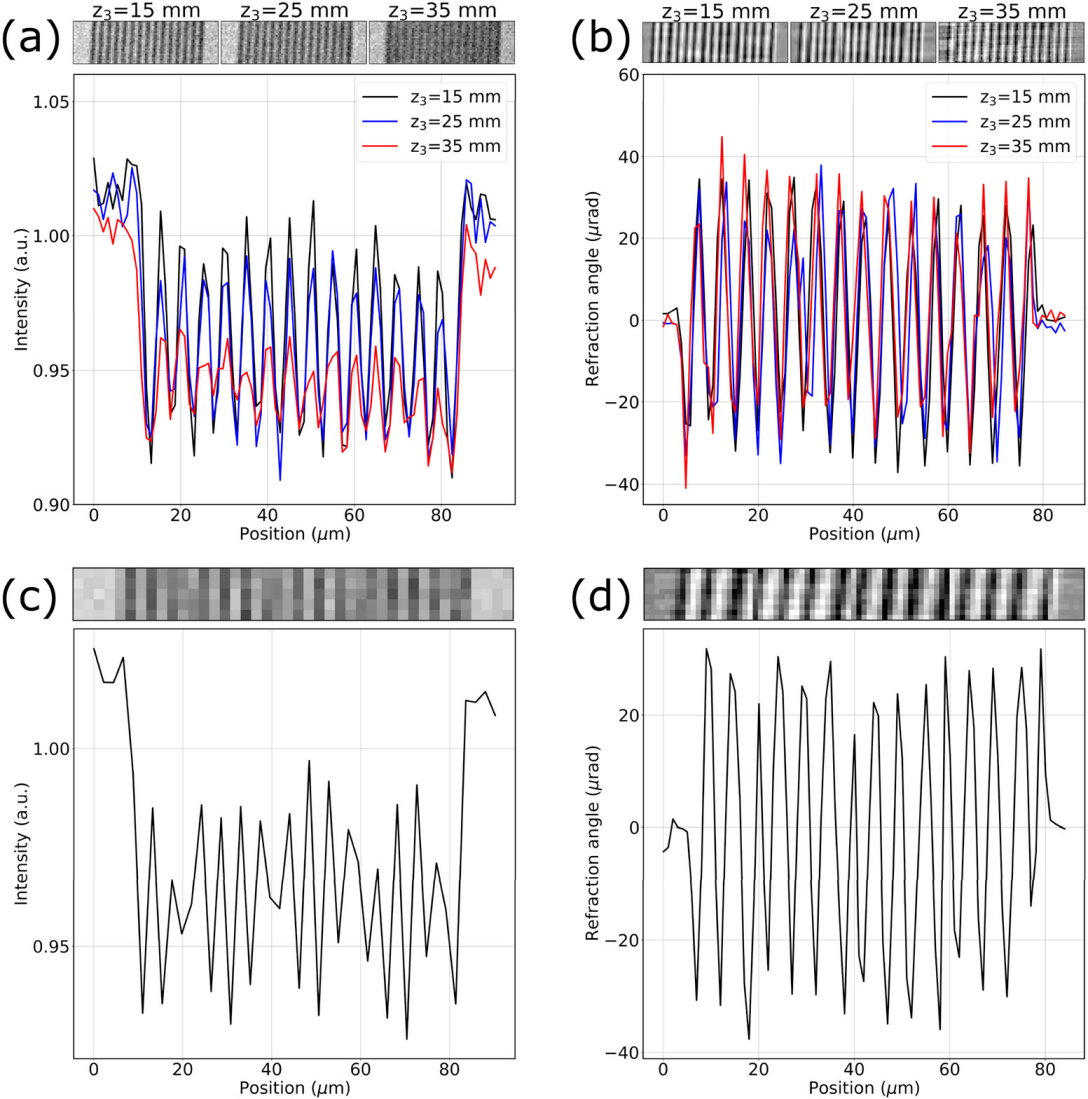
Images of a 2.5 *μ*m-wide bar pattern and intensity profiles measured in propagation-based (a) and beam tracking mode (b). Images were taken at a propagation distance (*z*_3_) of 15, 25, and 35 mm, corresponding to a projected focal spot size of 2.4, 4.0, and 5.6 *μ*m, respectively. The same bar pattern is also imaged with a 2 × 2 detector pixel binning at *z*_3_ = 15 mm and displayed with the corresponding intensity profiles in propagation-based (c) and beam tracking mode (d).

Similarly, images of the 2.5 *μ*m-wide bar pattern collected at the shortest sample-to-detector distance z_3_ = 15 mm are shown in Fig. 2 after a 2 × 2 detector pixel binning, resulting in an effective pixel size of 2.2 *μ*m, alongside their corresponding intensity profiles for both propagation-based (c) and beam tracking (d) modes. Binning of the beam tracking images was performed before retrieval, effectively emulating a detector with larger pixel pitch. While in propagation-based imaging increasing the pixel size leads to a loss of visibility in the intensity profile as well as to aliasing artifacts, the refraction signal is unaffected by the increase in pixel size. This result breaks the link between the factors that traditionally determine spatial resolution in the x-ray imaging system (focal spot size and detector pixel pitch)^16^ and the resolution level achieved in an image, provided that the setup is optimized so that beamlets can be adequately sampled and are independent from each other. Having demonstrated the independence of the beam tracking refraction signal from the focal spot size and detector pixels pitch, we further investigated the resolution limit in the three retrieved contrast channels—namely, transmission, refraction, and scattering, for varying sizes of the bar pattern features. Figure 3 shows images of the bar pattern for the three different bar sizes in transmission (a), refraction (e), and scattering (i) as well as intensity profiles for each channel and each feature size [panels (b)–(d), (f)–(h), and (j)–(l)]. All images were acquired at the same propagation distance *z*_3_ = 15 mm.

**FIG. 3.**
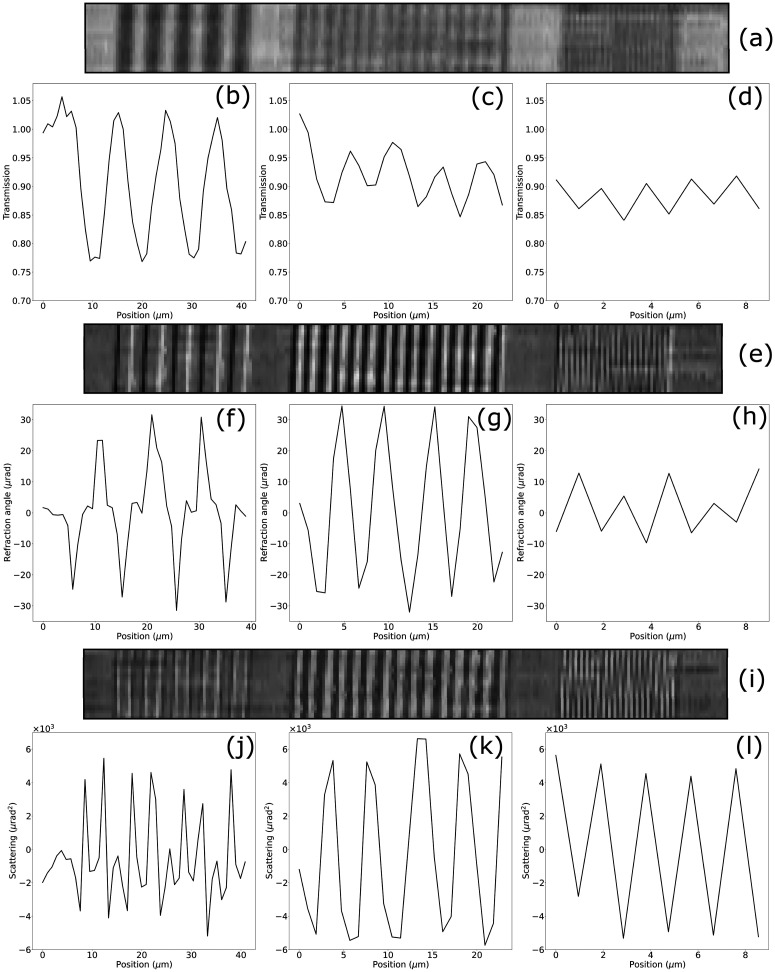
Retrieved images in the transmission (a), refraction (e), and scattering (i) channels of a bar pattern with varying feature sizes imaged at z_3_ = 15 mm. Intensity profiles in the three contrast channels are shown for the 5 *μ*m (b), (f), and (j), 2.5 *μ*m (c), (g), and (k) and 1 *μ*m (d), (h), and (l) feature size.

While the intensity profiles for the 5 and 2.5 *μ*m wide features show full modulation and quantitatively consistent values for both feature sizes in transmission, refraction, and scattering, transmission and refraction profiles show a reduced visibility for the 1 *μ*m-wide bar pattern, i.e., for a feature size smaller that the mask aperture. This result demonstrated how the spatial resolution in a beam tracking system is limited by the mask aperture size providing an experimental demonstration of the theoretical calculations reported in Ref. 17 for edge illumination.

The combination of the two findings derived so far is pivotal for the development of a multi-resolution system where it would be possible to investigate samples across different scales by changing the mask aperture without other modifications, e.g., reducing source focal spot size or detector pixel pitch.

An additional, unexpected observation is that the scattering signal appears to provide unreduced modulation centered in zero [see Fig. 3(l)], beyond the intrinsic resolution of the system given by the mask aperture. So far, this signal has been used to reveal the presence of unresolved microscopic structures of the sample;^5,18^ here, it is seen to also provide a possible means to resolve periodic structures below the system resolution, at least in the limited range explored in this experiment. If confirmed, this could provide a means to further extend the resolution limit of this imaging modality. Finally, from panels (j)*–*(l), it can be noted that both positive and negative scattering values are retrieved: a demonstration that the negative values are real (i.e., correspond to an actual reduction of the beamlets' width) is provided in the supplementary material.

As an example of biological samples, a dry beetle antenna and an equine tendon were imaged. The thin tendon section, excised postmortem, has been fixated in 4% paraformaldehyde and in 70% ethanol and imaged in an ethanol atmosphere using a polychromatic beam, i.e., after removing the monochromator in the setup of Fig. 1. Figure 4 shows the retrieved transmission (a) and (e), refraction (b) and (f), and scattering (c) and (g) images of the beetle antenna and tendon, respectively. The scattering images show a weak edge-enhancing signal but lacks areas where this signal specifically lights up, which is arguably due to the lack of features smaller than the aperture size. The complementarity of the transmission and refraction signals is shown in Figs. 4(h) and 4(i), where the articulation socket of the antenna as well as fibrous features of the tendon are enhanced in the refraction channel (cyan).

**FIG. 4.**
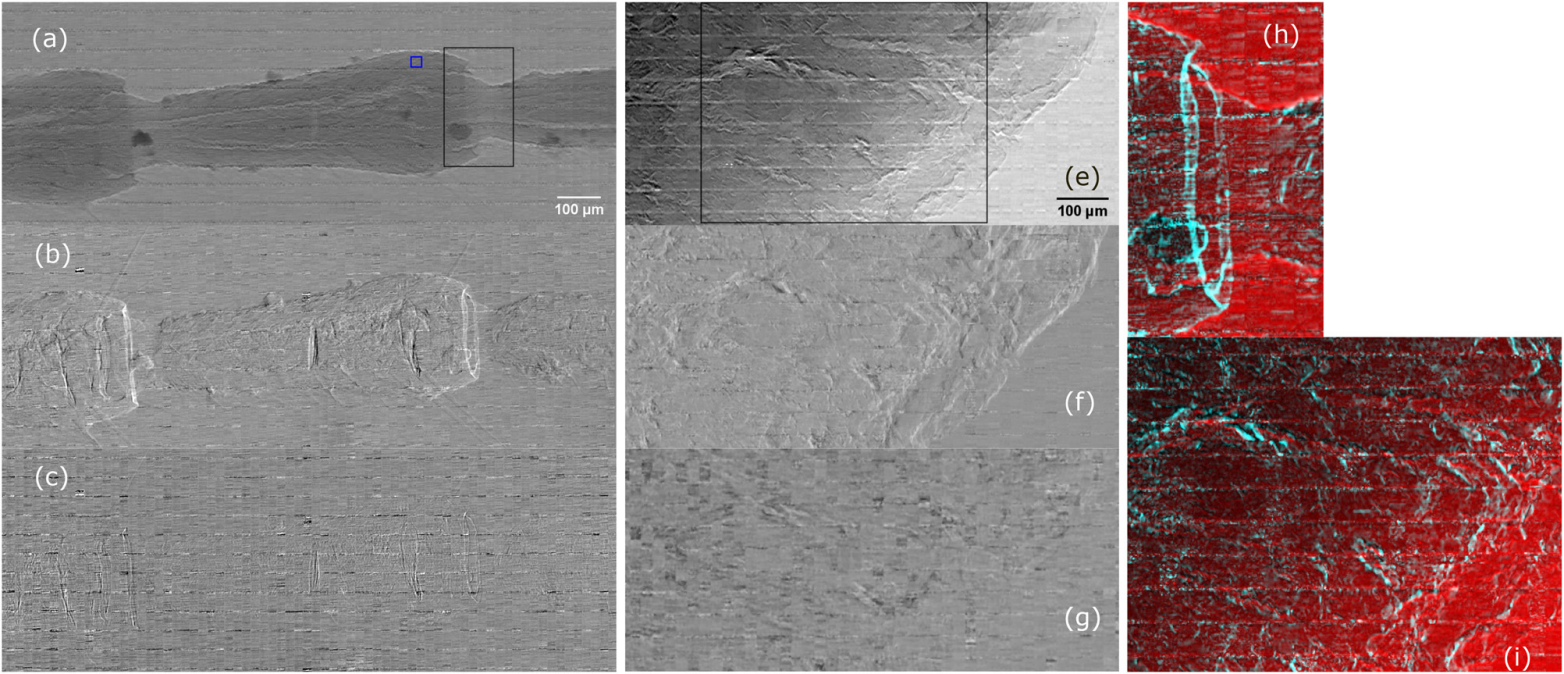
Absorption (a) and (e), refraction (b) and (f), and scattering (c) and (g) retrieved for a beetle antenna and equine tendon, respectively. Transmission and refraction for the region of interest (ROI) highlighted by a black square in panels (a) and (e) are displayed in (h) and (i) in red and cyan, respectively. Structured noise, oriented in the direction orthogonal to the mask slits, is visible in the retrieved images. This is due to the manufacturing process of the specific mask used in this work and should not be intended as an intrinsic limitation of the technique.

In this Letter, we demonstrated the instrument with a monochromatic beam, because of our current focus on quantitative imaging. However, we expect the approach to be applicable to polychromatic spectra: This has been demonstrated before for beam tracking,^10^ and indeed images in Figs. 4(e)–4(g) have been obtained with a polychromatic beam. A key challenge in terms of further developing polychromatic beam tracking in a microscopy context is the increased mask aspect ratio required to stop higher energy x rays: The partial transmission through the mask absorbing septa results in a higher background signal between beamlets, i.e., in a reduction in visibility, which, in turn, affects the imaging performance of the system as discussed in Ref. 13.

In conclusion, we presented a low-energy x-ray phase-based microscope for biological imaging and provided an experimental demonstration of the resolution limits of the system. These results pave the way for implementing multi-resolution and multi-contrast imaging through the use of masks with different aperture sizes, while relaxing the requirements in terms of focal spot size and detector pixel pitch. An ability of the scatter signal to maintain a high modulation also at spatial frequencies below the aperture-driven system resolution was also reported.

See the supplementary material for a detailed analysis of the modulation observed in the scattering signal for a bar pattern with feature sizes larger than the mask aperture width and a comparison with simulations.

## Data Availability

The data that support the findings of this study are available from the corresponding author upon reasonable request.

## References

[1] M. Endrizzi , Nucl. Instrum. Methods Phys. Res., Sect. A 878, 88 (2018). 10.1016/j.nima.2017.07.036

[2] S. W. Wilkins , T. E. Gureyev , D. Gao , A. Pogany , and A. W. Stevenson , Nature 384, 335 (1996). 10.1038/384335a0

[3] P. R. T. Munro , K. Ignatyev , R. D. Speller , and A. Olivo , Proc. Natl. Acad. Sci. 109, 13922 (2012). 10.1073/pnas.1205396109 22891301 PMC3435200

[4] A. Olivo and R. Speller , Phys. Med. Biol. 52, 6555 (2007). 10.1088/0031-9155/52/22/001 17975283

[5] M. Endrizzi , P. C. Diemoz , T. P. Millard , J. L. Jones , R. D. Speller , I. K. Robinson , and A. Olivo , Appl. Phys. Lett. 104, 024106 (2014). 10.1063/1.4861855

[6] I. Zanette , T. Zhou , A. Burvall , U. Lundström , D. Larsson , M. Zdora , P. Thibault , F. Pfeiffer , and H. Hertz , Phys. Rev. Lett. 112, 253903 (2014). 10.1103/PhysRevLett.112.253903 25014818

[7] F. Pfeiffer , T. Weitkamp , O. Bunk , and C. David , Nat. Phys. 2, 258 (2006). 10.1038/nphys265

[8] M. Töpperwien , F. van der Meer , C. Stadelmann , and T. Salditt , Proc. Natl. Acad. Sci. 115, 6940 (2018). 10.1073/pnas.1801678115 29915047 PMC6142271

[9] F. A. Vittoria , M. Endrizzi , P. C. Diemoz , U. H. Wagner , C. Rau , I. K. Robinson , and A. Olivo , Appl. Phys. Lett. 104, 134102 (2014). 10.1063/1.4870528

[10] F. A. Vittoria , G. K. N. Kallon , D. Basta , P. C. Diemoz , I. K. Robinson , A. Olivo , and M. Endrizzi , Appl. Phys. Lett. 106, 224102 (2015). 10.1063/1.4922189

[11] C. J. M. Jones , F. A. Vittoria , A. Olivo , M. Endrizzi , and P. R. T. Munro , Opt. Lett. 43, 3874 (2018). 10.1364/OL.43.003874 30106905

[12] L. Brombal , G. Kallon , J. Jiang , S. Savvidis , P. De Coppi , L. Urbani , E. Forty , R. Chambers , R. Longo , A. Olivo , and M. Endrizzi , Phys. Rev. Appl. 11, 034004 (2019). 10.1103/PhysRevApplied.11.034004

[13] M. Esposito , L. Massimi , I. Buchanan , J. D. Ferrara , M. Endrizzi , and A. Olivo , in *Medical Imaging 2022: Physics of Medical Imaging* ( SPIE, 2022), Vol. 12031, pp. 1–7. 10.1117/12.2609441 PMC9783294 36567972

[14] F. A. Vittoria , M. Endrizzi , P. C. Diemoz , A. Zamir , U. H. Wagner , C. Rau , I. K. Robinson , and A. Olivo , Sci. Rep. 5, 16318 (2015). 10.1038/srep16318 26541117 PMC4635357

[15] T. E. Gureyev , S. C. Mayo , D. E. Myers , Y. Nesterets , D. M. Paganin , A. Pogany , A. W. Stevenson , and S. W. Wilkins , J. Appl. Phys. 105, 102005 (2009). 10.1063/1.3115402

[16] T. E. Gureyev , Y. I. Nesterets , A. W. Stevenson , P. R. Miller , A. Pogany , and S. W. Wilkins , Opt. Express 16, 3223 (2008). 10.1364/OE.16.003223 18542410

[17] P. C. Diemoz , F. A. Vittoria , and A. Olivo , Opt. Express 22, 15514 (2014). 10.1364/OE.22.015514 24977810

[18] F. Pfeiffer , M. Bech , O. Bunk , P. Kraft , E. F. Eikenberry , C. Brönnimann , C. Grünzweig , and C. David , Nat. Mater. 7, 134 (2008). 10.1038/nmat2096 18204454

